# Absence of mutations in the coding sequence of the potential tumor suppressor 3pK in metastatic melanoma

**DOI:** 10.1186/1477-3163-4-23

**Published:** 2005-12-20

**Authors:** Roland Houben, Jürgen C Becker, Ulf R Rapp

**Affiliations:** 1 Institut für Medizinische Strahlenkunde und Zellforschung (MSZ), Universität Würzburg, Versbacher Str. 5, D-97078 Würzburg, Germany; 2 Hautklinik, Universität Würzburg, Josef Schneider Str.2, D-97078 Würzburg, Germany

## Abstract

**Background:**

Activation of Ras or Raf contributes to tumorigenesis of melanoma. However, constitutive Raf activation is also a characteristic of the majority of benign melanocytic nevi and high intensity signaling of either Ras or Raf was found to induce growth inhibition and senescence rather than transformation. Since the chromosome 3p kinase (3pK)) is a target of the Ras/Raf/Mek/Erk signaling pathway which antagonizes the function of the oncogene and anti-differentiation factor Bmi-1, 3pK may function as a tumor suppressor in tumors with constitutive Ras/Raf activation. Consequently, we tested whether inactivating 3pK mutations are present in melanoma.

**Methods:**

30 metastatic melanoma samples, which were positive for activating mutations of either BRaf or NRas, were analyzed for possible mutations in the *3pk *gene. The 10 coding exons and their flanking intron sequences were amplified by PCR and direct sequencing of the PCR products was performed.

**Results:**

This analysis revealed that besides the presence of some single nucleotide polymorphisms in the *3pk *gene, we could not detect any possible loss of function mutation in any of these 30 metastatic melanoma samples selected for the presence of activating mutations within the Ras/Raf/Mek/Erk signaling pathway.

**Conclusion:**

Hence, in melanoma with constitutively active Ras/Raf inactivating mutations within the *3pk *gene do not contribute to the oncogenic phenotype of this highly malignant tumor.

## Background

Transcriptional repressors of the Polycomb group (PcG) are part of the cellular epigenetic machinery [1]. The PcG protein Bmi-1 was described as an oncogene in murine lymphomas, which acts via repression of the ink4A locus [2]. Its implication in human tumorigenesis is suggested by the finding that it is amplified or overexpressed in several human cancers [3-5] Bmi-1 was shown to be an anti-differentiation factor, e.g. its overexpression extents the replicative life span of human fibroblasts by suppressing the p16-dependent senescence pathway [6]. In addition, Bmi-1 is a self-renewal factor for hematopoietic and neural stem cells [7-9].

The gene of the chromosome 3p kinase (3pK; also known as Mapkap Kinase 3) was isolated by analysis of the chromosomal region 3p21.3 which is homozygously deleted in two cancer cell lines [10]. Very recently we found that 3pK interacts with and phosphorylates Bmi-1 thereby diminishing Bmi-1 chromatin association [11]. Overexpression of 3pK has growth suppressive properties in various cell lines, which can be overcome by Bmi-1 overexpression (Voncken and Rapp, unpublished observation). It should be noted, that 3pK is a target of the classical mitogenic Ras/Raf/Mek/Erk signaling pathway and is activated by phosphorylation through Erk [12].

It is well established that activation of the Ras/Raf/Mek/Erk pathway contributes to tumorigenesis and melanoma is the tumor with the highest prevalence of activating mutations within the *braf *gene [13-15]. On the other hand activating BRaf mutations are present in the majority of benign melanocytic lesions and high intensity signaling of either Ras or Raf were found to induce growth inhibition and senescence rather than transformation [16]. These observations may be explained by an activation of the 3pK/Bmi-1/Ink4A branch. Thus, inactivating 3pK mutation may be part of the neoplastic transformation of tumors with activated Ras or Raf such as melanoma.

## Materials and methods

### Tumor material

Paraffin embedded tumor samples from 30 metastatic melanomas were obtained by surgical excision. All tumors have undergone routine histology for diagnosis. The immediately adjacent slides from the blocks were used for DNA extraction. Informed consent was obtained from all patients prior to any of these measures.

### Polymerase chain reaction

Genomic DNA was isolated from paraffin embedded tumor samples using a DNA Isolation Kit (Qiagen). Applying conventional PCR on these templates let to frequent drop outs not yielding the desired product. We therefore applied Nested PCR protocols with a first 22 cycle PCR reaction (30 μl) followed by a second 35 cycle PCR reaction (55 μl) in which 1 μl of the first reaction served as template. The Cycles always consisted of 30 seconds denaturing at 95°C, 30 seconds annealing followed by 30 seconds elongation at 72°C. Primers and corresponding annealing temperatures are given in table 1.

**Table 1:** Conditions of the nested PCR for the 10 coding exons of the 3pK gene. Primer sequences and the corresponding annealing temperatures are given.

**Exon**	**Primer**	**Annealing**
**2**	first PCR: ctcgcagcccagcccagttcag gctacgcctcagtttccccatct	66°C
	nested PCR: tctgggcgggactcactcttc gctacgcctcagtttccccatct	66°C
**3**	first PCR: ggcctcagagcttatatgtttttg gatgccgagggggtgtggag	63°C
	nested PCR: ggcctcagagcttatatgtttttg gtttcttgcccacccttgag	63°C
**4**	first PCR: ggcaggggcaagggtagg agatgggaagactgagaggaaca	64°C
	nested PCR: ctgaagcctgggcctatgatttg agatgggaagactgagaggaaca	64°C
**5**	first PCR: ttgtgctggcctgttagactgtgt aggctgggtgagaatgaaatgaga	67°C
	nested PCR: cccgggatagagaacctggatag aggctgggtgagaatgaaatgaga	67°C
**6**	first PCR: aggcccgggtctttttat cccacgtcacccgcctccat	66°C
	nested PCR: cacctgccttgcttccccttttgt cccacgtcacccgcctccat	58°C
**7**	first PCR: ctttctcctcccccaacttcaca cccaggcacatcccaggaat	66°C
	nested PCR: ctttctcctcccccaacttcaca ctccggactcccaggcacatc	66°C
**8**	first PCR: ggagtccggagggctggtattatt gaggctcaagaaggttagg	59°C
	nested PCR: actgctcccaccctatgccaaatg gaggctcaagaaggttagg	59°C
**9**	first PCR: ggctgccctgtatgagttatct gcagtgacaaagccaggtt	63°C
	nested PCR: ggctgccctgtatgagttatct aaagccaggttgtaggtgagg	63°C
**10**	first PCR: gtcccaggtgcctccagtttctaa aagccccgtttcattgagga	60°C
	nested PCR: ggagtagggggaaccagtgctgtc aagccccgtttcattgagga	64°C
**11**	first PCR: gggggtgatggttcctaagg cctcactcgggccaaccta	64°C
	nested PCR: tgggcacagaggcagcacagg cctcactcgggccaaccta	64°C

### Sequencing

Samples were sequenced using BigDye™ Terminator Cycle Sequencing Ready Reaction Kit (Applied Biosystems) according to the manufacturer's manual, and analyzed on an ABI PRISM 3100 Avant Genetic Analyzer. The sequences were first analyzed by visual inspection, looking for double peaks or untypical background signals. Additionally alignments with the published genomic sequence were performed using the DNAStar™ software.

## Results and discussion

Previously we had analyzed a series of 200 melanoma samples for the presence of activating mutations in BRaf and NRas [17]. Out of these samples we choose a set of 30 metastatic melanomas of which 25 carried a BRaf (V599E, V599R or V599K) and 5 an NRas mutation (Q61R or Q61K). For these 30 samples we amplified the coding plus adjacent intron sequences of the exons 2 to 11 and subjected them to sequence analysis. To this end, no nucleotide exchange which would alter the amino acid sequence of the 3pK protein was detected indicating that the 3pKinase is not frequently affected by mutations in metastatic melanoma with constitutively activated Ras/Raf signaling pathway.

**Figure 1:**
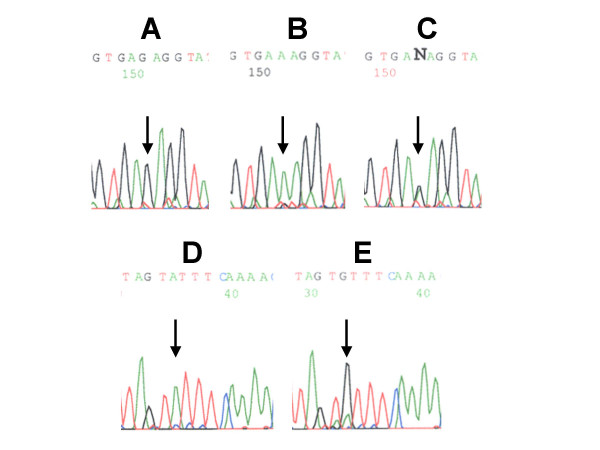
**Sequence chromatographs of intronic sequences from the 3pK amplicons exon 4 (A, B and C) an exon 9 (D and E)**. 42 nucleotides downstream of exon 4 either homozygously G or homozygously A or a heterozygous genotype was found. (A, B and C). 160 nucleotides upstream of exon 9 we found predominantly an A instead of the published G (D). Only in one case signals for G and A were detectable. (E).

An intronic single nucleotide polymorphism (SNP) was detected 42 nucleotides downstream (in 3' direction) of exon 4. Within the sequence GCAGGAGGATTCAGGGTGAG the last G was frequently altered to A. In two cases we received only an A signal. In 13 cases we found heterozygous A and G and in 15 cases only the published G was present (Figure 1A, B and 1C). 160 nucleotides upstream (in 5' direction) of exon 9 within the sequence GTTTCAAAACAAGAAATAG the first G was altered to an A in all but one case (Figure 1D). Only in this one sample in addition to the A signal a signal for a G was detectable (Figure 1E). Because of the very rare appearance of the published sequence we asked whether this alteration might be a melanoma specific SNP. Therefore we amplified and sequenced this region from genomic DNA of 14 healthy donors. In all 14 cases we found G instead of A in the corresponding position, demonstrating that this is the preferential nucleotide in the german population.

The absence of inactivating mutations within the *3pk *gene, however, does not rule out the possibility that 3pK function in these tumors might be affected by reduced expression levels. For example, in uveal melanoma mutations in the coding sequence of the tumor suppressor Ink4A are very rare, but hypermethylation of its promoter is a common cause of reduced Ink4a expression in these tumors [18].

In a previous study we correlated the Ras/Raf mutational status of 200 melanoma lesions with the the clinical course; the presence of activating mutations within primary tumors did not impact the prognosis – a notion further substantiated by the results of multivariate analysis which revealed that for this patient cohort mutations in the *braf *gene were not correlated with established markers for bad prognosis such as tumor thickness or ulceration. The presence of BRaf mutations in metastatic lesions, however, was associated with poor prognosis, i.e. shortened survival from either the removal of the respective metastases or clinical diagnosis of stage IV disease. Because of this observation together with the fact that carcinogenesis is a multistage process one can assume that additional genetic or epigenetic alterations are necessary for the neoplastic transformation of melanocytic cells and that in the absence of these alteration(s) the poor prognosis properties of activated BRaf are masked. In the present study, we ruled out, that inactivating mutations of 3pk are frequent events in metastatic melanoma with constitutively activated Ras/Raf signaling pathway. Others tested a possible tumor suppressor function of 3pK by ectopic expression of 3pk in two cancer cell lines. This however did not antagonize the tumorous growth of these cells in Scid mice [19]. Therefore 3pK remains a canditate tumor suppressor and its possible role in tumor development needs further investigation.

## Authors' contributions

RH performed the mutation analysis. RH and JCB wrote the manuscript. JCB provided the gDNA from the tumor samples. UR was the supervisor of the project and designed the study.

All authors read and approved the final manuscript.
